# Serum miRNAs Support the Indication for MRI-Ultrasound Fusion-Guided Biopsy of the Prostate in Patients with Low-PI-RADS Lesions

**DOI:** 10.3390/cells10061315

**Published:** 2021-05-25

**Authors:** Bastian Keck, Angelika Borkowetz, Julia Poellmann, Thilo Jansen, Moritz Fischer, Susanne Fuessel, Andreas Kahlmeyer, Manfred Wirth, Johannes Huber, Alexander Cavallaro, Matthias Hammon, Ivan Platzek, Arndt Hartmann, Gustavo Baretton, Frank Kunath, Danijel Sikic, Helge Taubert, Bernd Wullich, Kati Erdmann, Sven Wach

**Affiliations:** 1 Department of Urology and Pediatric Urology, University Hospital Erlangen, Friedrich-Alexander Universität Erlangen, Krankenhausstrasse 12, 91054 Erlangen, Germany; bastian.keck@web.de (B.K.); julia.poellmann@uk-erlangen.de (J.P.); thilo.jansen@googlemail.com (T.J.); moritzfischer2001@yahoo.de (M.F.); andreas@dr-kahlmeyer.de (A.K.); frank.kunath@uk-erlangen.de (F.K.); danijel.sikic@uk-erlangen.de (D.S.); Bernd.Wullich@uk-erlangen.de (B.W.); sven.wach@uk-erlangen.de (S.W.); 2 Department of Urology, Medical Faculty Carl Gustav Carus, Technische Universität Dresden, Fetscherstrasse 74, 01307 Dresden, Germany; Angelika.Borkowetz@uniklinikum-dresden.de (A.B.); Susanne.Fuessel@uniklinikum-dresden.de (S.F.); Manfred.Wirth@uniklinikum-dresden.de (M.W.); Johannes.Huber@uniklinikum-dresden.de (J.H.); Kati.Erdmann@uniklinikum-dresden.de (K.E.); 3 Comprehensive Cancer Center Erlangen-EMN (CCC ER-EMN), Östliche Stadtmauerstrasse 30, 91054 Erlangen, Germany; alexander.cavallaro@uk-erlangen.de (A.C.); arndt.hartmann@uk-erlangen.de (A.H.); 4 Member of the Association of Scientists in Urological Research (UroFors) of the German Society of Urology, Martin-Buber-Straße 10, 14163 Berlin, Germany; 5 Department of Radiology, University Hospital Erlangen, Friedrich-Alexander Universität Erlangen, Maximiliansplatz 3, 91054 Erlangen, Germany; matthias.hammon@gmail.com; 6 Department of Radiology and Interventional Radiology, Medical Faculty Carl Gustav Carus, Technische Universität Dresden, Fetscherstrasse 74, 01307 Dresden, Germany; Ivan.Platzek@uniklinikum-dresden.de; 7 Institute of Pathology, University Hospital Erlangen, Friedrich-Alexander Universität Erlangen, Krankenhausstrasse 8-10, 91054 Erlangen, Germany; 8 Institute of Pathology, Medical Faculty Carl Gustav Carus, Technische Universität Dresden, Fetscherstrasse 74, 01307 Dresden, Germany; Gustavo.Baretton@uniklinikum-dresden.de; 9 National Center for Tumor Diseases (NCT), Fetscherstrasse 74, 01307 Dresden, Germany

**Keywords:** diagnosis, microRNA, miRNA, mpMRI, PI-RADS, prostate biopsy, prostate cancer

## Abstract

Multiparametric MRI (mpMRI) and targeted biopsy of the prostate enhance the tumor detection rate. However, the prediction of clinically significant prostate cancer (PCa) is still limited. Our study tested the additional value of serum levels of selected miRNAs in combination with clinical and mpMRI information for PCa prediction and classification. A total of 289 patients underwent targeted mpMRI-ultrasound fusion-guided prostate biopsy complemented by systematic biopsy. Serum miRNA levels of miRNAs (miR-141, miR-375, miR-21-5p, miR-320b, miR-210-3p, let-7c, and miR-486) were determined by quantitative PCR. Detection of any PCa and of significant PCa were the outcome variables. The patient age, pre-biopsy PSA level, previous biopsy procedure, PI-RADS score, and serum miRNA levels were covariates for regularized binary logistic regression models. The addition of miRNA expression of miR-486 and let-7c to the baseline model, containing only clinical parameters, increased the predictive accuracy. Particularly in patients with PI-RADS ≤3, we determined a sensitivity for detecting significant PCa (Gleason score ≥ 7a corresponding to Grade group ≥2) of 95.2%, and an NPV for absence of significant PCa of 97.1%. This accuracy could be useful to support patient counseling in selected cases.

## 1. Introduction

Multiparametric magnetic resonance imaging (mpMRI) and targeted biopsy of tumor-suspicious lesions have become established diagnostic tools for the detection of prostate cancer (PCa) [1], since ultrasound-guided biopsy alone misses approximately 25–30% of PCa cases [2,3]. MpMRI has demonstrated a higher sensitivity and specificity for the detection of PCa [4] and is widely accepted among healthcare practitioners [5].

An increasing amount of published data has shown that targeted MRI-ultrasound fusion-guided biopsies have the potential to reduce the diagnosis of insignificant PCa and to enhance the detection rate of clinically significant PCa while reducing the number of biopsies, as the Prostate Imaging Reporting and Data System (PI-RADS) has a good diagnostic accuracy and correlation with PCa aggressiveness [6,7]. Clinical parameters, such as the pre-biopsy serum PSA level and mpMRI of the prostate, represent the basis of the clinical information used for decision making and patient counseling.

Nevertheless, in clinical practice, the diagnosis of PCa is accompanied by various uncertainties. Therefore, additional factors, not only enhancing the prediction of tumor presence but also tumor classification, are an important clinical need. We have previously shown that miRNAs are consistently deregulated in PCa and, by regulating their cognate protein targets, contribute to prostatic carcinogenesis [8,9]. Moreover, we and others could demonstrate that miRNAs derived from whole blood [10] or blood serum [11,12,13,14] can serve as biomarkers with the potential to differentiate PCa patients from patients with benign prostatic hyperplasia. However, it is not fully understood whether different miRNAs originate from tumor cells or if they are rather a response of the host organism to the presence of PCa [15]. Therefore, we set up a prospective MRI-guided biopsy study supplemented with serum-miRNA-analysis to evaluate the additional predictive value of the miRNA analysis to the clinical factors. Hereby, the addition of serum-based miRNA analysis to the clinical routine factors was able to enhance the predictive values of the mathematical models for predicting significant PCa.

## 2. Materials and Methods

### 2.1. Study Population

For the discovery cohort, a total of 80 consecutive patients with suspicion of PCa were recruited between January 2015 and July 2016. Patients were referred to the University Hospital Erlangen for targeted MRI-ultrasound fusion-guided prostate biopsy, which was complemented by systematic biopsy. Prostate mpMRIs were performed on 3T devices (Siemens Medical Solutions, Erlangen, Germany). All image sets were examined by an experienced radiologist and were scored according to PI-RADS V2.0. A combined targeted and systematic 12-core transrectal MRI-ultrasound fusion-guided biopsy was performed using the General Electric LOGIQ E9 (GE Healthcare, Solingen, Germany) system. Suspicious regions (PI-RADS scores 3, 4, 5) were biopsied specifically and non-suspicious regions (PI-RADS scores 1, 2) systematically. Written informed consent was obtained before biopsy and the study was performed according to the Declaration of Helsinki. Ethical approval was provided by the ethics institutional review board of the University Hospital Erlangen (No. 3755, dated February 2008).

For the validation cohort, a total of 209 consecutive patients with suspicion of PCa were recruited between January and December 2015. Patients were referred to the University Hospital Dresden for targeted MRI-ultrasound fusion-guided prostate biopsy. Prostate mpMRIs were performed on 3T devices (Siemens Medical Solutions, Erlangen, Germany). All image sets were examined by two experienced radiologists and scored according to PI-RADS V.2.0. At this study site, the BioJet-System (d&k Technologies, Barum, Germany) was used for MRI-ultrasound fusion-guided biopsy as described previously [16]. Briefly, fusion-guided prostate biopsy was performed in a transperineal approach, taking at least two cores per lesion. Lesions classified as PI-RADS ≥2 were biopsied in a targeted fashion. Subsequently, every patient underwent a transrectal 12-core systematic biopsy. Written informed consent was obtained before biopsy. Ethical approval was provided by the ethics institutional review board of the Technische Universität Dresden (No. EK194092004, dated July 2009).

According to the EAU guidelines, both diagnostic procedures are regarded as equal in their diagnostic performance [17]. The clinical end points examined were histologically confirmed PCa and clinically significant PCa (csPCa), defined as Gleason score ≥7a (corresponding to Grade group ≥2). We followed a holistic approach with histologic diagnosis as the final outcome. In clinical practice, it is not relevant if significant PCa was found in a targeted or non-targeted biopsy core.

### 2.2. Blood Sampling and RNA Isolation

Before biopsy, venous blood was drawn into coagulation tubes (Sarstedt, Nümbrecht, Germany) and further processed within two hours. Serum was prepared from the coagulated blood by centrifugation (2000 g for 10 min) and samples were stored in aliquots at −80 °C. Serum miRNAs were prepared from 200 µL of serum with the miRCURY RNA Isolation Kit for biofluids (Exiqon, Vedbaek, Denmark) in the discovery cohort and the miRNA plasma kit (Promega, Madison, WI, USA) using an automated Maxwell RSC device (Promega) in the validation cohort, according to the respective manufacturer’s recommendations.

### 2.3. Quantitative PCR

Synthesis of cDNA and subsequent quantification of miRNAs was conducted using TaqMan reverse transcription reagents and miRNA-specific TaqMan microRNA Expression Assays for miR-21-5p (ID: 000397), miR-141-3p (ID: 000463), miR-210-3p (ID: 000512), miR-320b (ID: 002844), miR-375-3p (ID: 000564), miR-486-5p (ID: 001278) let-7c-5p (ID: 000379), and miR-16-5p as reference (ID: 000391) (Thermo Scientific, Darmstadt, Germany) according to the manufacturer’s recommendations. Briefly, a constant volume of 4 µl of isolated serum RNA was reverse transcribed in a total volume of 15 µl using a customized pool of the respective miRNA-specific stem-loop primers and the TaqMan MicroRNA Reverse Transcription Kit (Thermo Scientific). The resulting cDNA was pre-amplified for 12 cycles using pooled miRNA-specific primer-probe sets and the TaqMan PreAmp Mastermix (Thermo Scientific). Quantitative PCR reactions were performed in a LightCycler 480 Real-Time PCR System (Roche Diagnostics, Mannheim, Germany) in a total volume of 10 µL containing 1 µL of pre-amplified cDNA (1:5 pre-diluted), 0.5 µL of miRNA specific primer-probe sets, 5 µL of the GoTaq probe qPCR master mix (Promega) and 3.5 µL of nuclease-free water. All reactions were measured in duplicate with the following conditions: initial denaturation at 95 °C for 10 min followed by 45 cycles at 95 °C for 15 s and 60 °C for 60 s. Threshold cycles (Ct) were determined by the second derivative method and then averaged. For relative quantification, every sample was analyzed for the endogenous reference miRNA miR-16-5p. Baseline and threshold settings were constant across the complete experimental series. Reactions were regarded as valid when the threshold cycle Ct of miR-16-5p was within the range of 13–23. Expression levels were calculated by applying the ΔCt method [18], given as the ΔCt between reference and test miRNA. By using the normfinder script, we could confirm that in our patient cohort, miR-16-5p gene expression is stable enough to be considered as a reference gene (normfinder stability value 0.08). Any reactions that did not show any amplification were manually set to Ct 40 if the Ct of the reference miRNA miR-16-5p of the respective sample was within the range of 13–23. All reactions were performed blinded to the study end points.

### 2.4. Statistical Methods

Differences in the clinical factors and serum miRNA expression values were analyzed using chi-square (factor variables) or nonparametric Mann–Whitney (continuous variables) statistical tests. The predictive modeling was performed using regularized generalized logistic regression modeling as implemented in the glmnet package for R. A fitted probability of 50% was used as the cut-off for assigning the binomial group labels. Receiver operator characteristics (ROC) were calculated using the pROC package. For the estimation of the benefit of predictive models we used the decision curve analysis method [19]. The net expected regret difference and individual thresholds of 20 clinical urologists were calculated based on the regret theory as described [20]. To determine reference gene stability, we used the normfinder script [21]). All calculations were performed with the R statistical framework Ver. 3.2.1 (R Foundation for Statistical Computing, Vienna, Austria. http://www.R-project.org/).

## 3. Results

We acquired a total of 289 patients consisting of a discovery cohort (*n* = 80) and a validation cohort (*n* = 209) undergoing targeted MRI-ultrasound fusion-guided prostate biopsy. The patients’ characteristics and univariate association of the characteristics with the clinical end points PCa and significant PCa with a Gleason score ≥7a are summarized in Table 1; a detailed summary of tumor diagnosis within PI-RADS lesions is shown in Supplementary Table S1. We have found csPCa both in systematic and in targeted biopsies. There were differences in the distribution of the number of patients undergoing primary or repeat MRI-ultrasound fusion-guided biopsy and the distribution of PI-RADS scores (both *p* < 0.001; chi-squared test) between the discovery and the validation cohort, whereas patient age, pre-biopsy PSA level, PCa detection rates, and the distribution of biopsy Gleason scores were not significantly different between the two patient cohorts.

As part of a variable selection process, we tested whether individual miRNAs are able to improve the accuracy of tumor prediction, when introduced into a regularized binary logistic regression model. One miRNA, miR-21-5p, failed to pass our internal quality control during data pre-processing and thus was omitted from further analyses.

In the discovery cohort (*n* = 80), two patients who were tumor-negative in the mpMRI-ultrasound fusion-guided biopsy already had an earlier diagnosis of PCa and were therefore regarded as tumor-positive in this analysis. Each miRNA was added separately to the baseline model and model characteristics were determined (Table 2; upper part). Four out of the seven miRNAs (miR-210-3p, miR-375-3p, miR-486-5p, and let-7c-5p) provided an advantage over the baseline model, quantified by either a gain in PPV/NPV or an increase in the area under the ROC curve (AUC).

We then applied the established regression models to the validation cohort (*n* = 209), and observed that all four identified miRNAs (miR-210-3p, miR-375-3p miR-486-5p, and let-7c-5p) also resulted in a gain in predictive accuracy in the independent validation cohort (Table 2; lower part).

Next, we tested the classification capabilities of the multivariate predictive model. The model consisting of clinical variables (patient age, pre-biopsy PSA level, previous biopsy procedure, and highest PI-RADS score) and miRNAs (miR-210-3p, miR-375-3p, miR-486-5p and let-7c-5p) achieved a PPV of 70.9% and an NPV of 68.6%. Detailed information about the model and the predictive performance is provided in Supplementary Table S2. The calibration plot of the regression model is shown in Supplementary Figure S1.

Finally, we explored the possibility to utilize clinical information and miRNA expression values to construct a binary regression model to discriminate between tumor-free or insignificant (Gleason score 6 corresponding to Grade group 1) and csPCa. This information might become especially useful in patients with lesions of PI-RADS ≤3 when clinicians have to decide whether to perform a biopsy or not. Two patients with confirmed PCa had a tumor-free biopsy result and were excluded from this analysis. We focused this approach on the patient subgroup with lesions of PI-RADS 1, 2, or 3, resulting in a final cohort size of 89. It is noteworthy that 21 of these patients were diagnosed with a csPCa of Gleason score ≥7a. Moreover, we noticed that two miRNAs (miR-210-3p and miR-375-3p) were present at rather low levels in patient serum, thereby resulting in a considerable number of “undetected” results. Therefore, in order to define a miRNA panel with a potential applicability in routine laboratory practice, we decided to further reduce the incorporated miRNA markers. The final predictive model was constructed using 3 clinical variables (patient age, pre-biopsy PSA level, and previous biopsy procedure) and two miRNAs (miR-486-5p and let-7c-5p). 

The highest Youden index was determined at a cut-off of 14%. To determine if this cut-off value of 14% represents a clinically reasonable threshold value, we interviewed 20 clinical decision makers and calculated individual threshold cut-off values [20] based on personal experience. The median of individual threshold values was 12% (interquartile range 8–28%). This demonstrated that our predictive model is able to provide additional information within a range of threshold values that are applicable in clinical practice.

**Figure 1:**
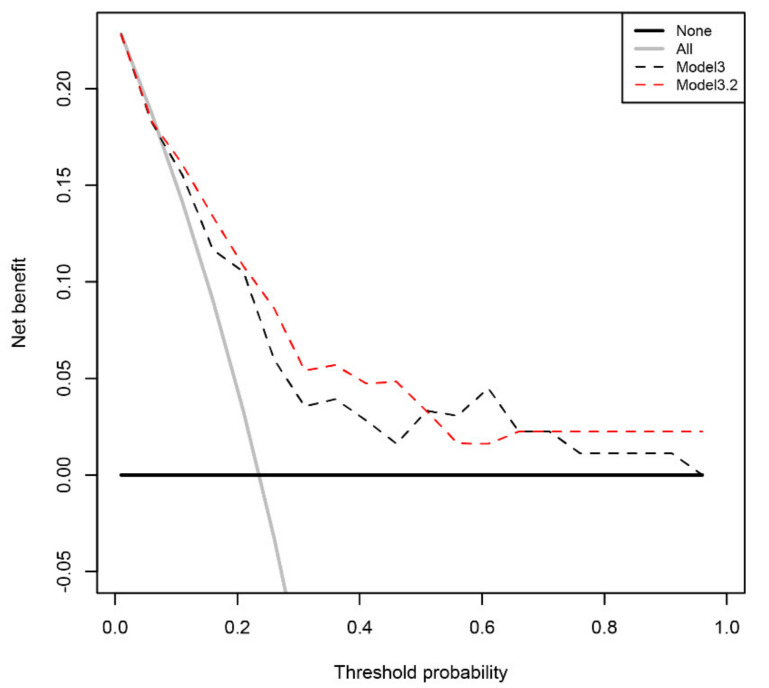
Decision curve analysis. The net benefit of using the mathematical models for biopsy indication instead of the extreme strategies is shown. Model3 includes PSA level, patient age and previous biopsy status, model3.2 includes PSA level, patient age, previous biopsy status, and the expression levels of miR-486 and let-7c. Model3.2 provides a benefit over the extreme strategies of recommending a biopsy in all patients or recommending a biopsy for no patient within a threshold range of 7% to 97%.

**Figure 2:**
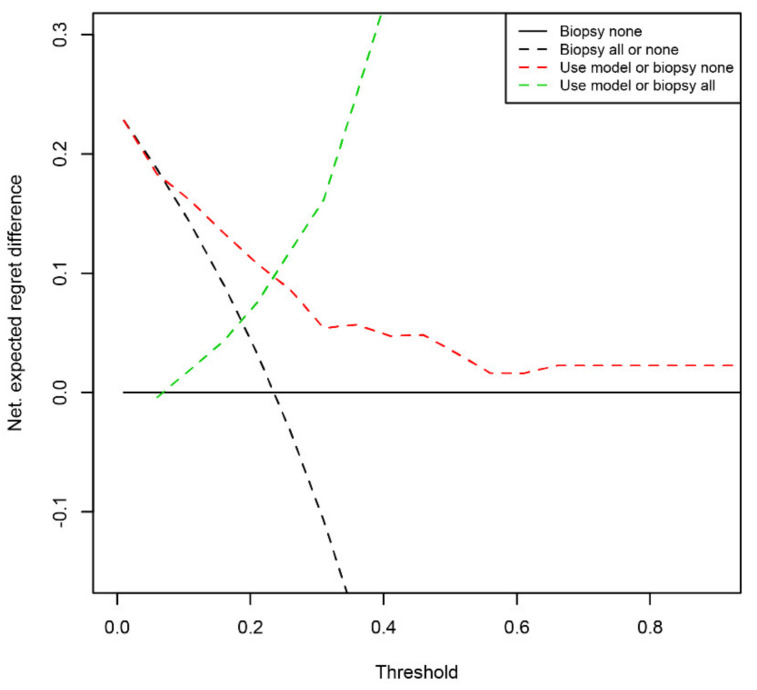
Expected Regret Difference. The net Expected Regret Difference of using the model3.2 for biopsy indication in pair-wise comparison. Within a threshold range of 7% and 97%, our model provided a benefit over both extreme strategies.

By classifying at a cut-off value of 14%, a total of 20 of the 21 csPCa cases were correctly identified. This high sensitivity of 95.2% of csPCa cases identified was accompanied by an NPV of 97.1% for the absence of csPCa. Detailed information about the predictive mathematical model and the predictive performance is provided in Table 3. The calibration plot of the regression model is shown in Supplementary Figure S2 and a receiver–operator curve comparison with the baseline model is given in Supplementary Figure S3. A decision curve analysis was used to quantify the benefit of applying the predictive model. At the specified threshold of 14% risk, the net benefit of the model accounts to 16.1% (Figure 1). Following a related method, the regret theory, we sought to more exactly determine the range of threshold values where our predictive model might provide a benefit for patients. Following the published equations [20], we calculated the Net Expected Regret Difference. Up to a threshold cut-off of 6%, our model does not provide a benefit over the strategy of biopsy all patients and above a cut-off of 98%, it does not provide a benefit over the strategy of biopsy no patient. Between 7% and 97%, our model provided a benefit over both extreme strategies (Figure 2 and Supplementary Table S3).

## 4. Discussion

A recent nationwide survey among clinicians in Germany underscores the widespread acceptance of mpMRI and targeted prostatic biopsies [5]. Despite the gain in diagnostic accuracy by mpMRI, there is still an on-going effort to utilize this information for risk classification. However, the combination of clinical parameters and mpMRI is a promising tool to reduce unnecessary prostate biopsies [22]. Here, we tested the additional predictive value of miRNAs incorporated in a regression model to predict PCa detection and tumor aggressiveness prior to mpMRI-ultrasound fusion-guided biopsy. MiRNAs were selected because of their differential expression in PCa tissue compared to non-malignant prostatic tissue [8,9]. Our study shows that it is possible to enhance the prediction of PCa detection by adding serum miRNA expression levels to clinical data and the PI-RADS score. However, up to now, there is a strong suggestion that PI-RADS 3 lesions should also be biopsied [5,23], because they might harbor significant PCa ranging from 12% [24] to 34% in current cohorts [25], even though other studies show only a limited incidence of significant PCa [26]. Yang et al. utilized mpMRI imaging information and routine clinical parameters for modeling cancer risk particularly in patients with PI-RADS 3 lesions and suggested their model for the reduction of unnecessary biopsies [27]. Interestingly, our mathematical models performed very well at identifying patients with csPCa of Gleason score ≥7a especially in the subgroup of patients with lesions of PI-RADS ≤3. We could identify patients with csPCa with a sensitivity of 95.2% while at the same time predicting the absence of csPCa with an NPV of 97.1% These properties of the model might be of high interest for clinicians to identify those patients that can be spared a prostate biopsy (high NPV) while still being able to detect the majority of significant PCa cases (high sensitivity).

Interestingly, none of the incorporated serum-miRNAs was significantly associated with PCa diagnosis or Gleason score by itself, although all the measured miRNAs have been described to be differentially expressed in PCa tissue and are suggested to play a role in prostatic carcinogenesis [8,9,12,28,29,30,31]. Moreover, we showed in previous studies that clinical parameters, such as the pre-biopsy PSA level, Gleason score, or TNM status, have only a limited impact on the overall abundance of miRNAs in the patients’ blood [10].

Molecular tests, such as the PCA3 test and others, have the potential to enhance such predictive models. Fenstermaker et al. demonstrated that a positive PCA3 test was associated with PCa detection in MRI-ultrasound fusion-guided targeted biopsies in patients with visible lesions in the primary setting of PCa detection [32]. However, the impact in highly suspicious lesions was limited in their study. They propose to test the value of the PCA3 test particularly in patients with low or equivocal PCa probability after mpMRI. With the proposed cut-off of 35, the PCA3 test exhibited a sensitivity of 62% and 73% as well as a NPV of 88% and 91% in patients with lesions of PI-RADS 2 and 3, respectively [32]. In the setting of primary, not mpMRI fusion-supported prostate biopsies, several studies evaluated molecular tests such as the PCA3 test or the prostate health index (PHI) and their abilities to predict the diagnostic outcome. Thereby, the PHI performed better than PSA alone with sensitivities ranging from 74–94% and specificities ranging from 49–72% [33,34]. In future studies, prostate volume, PSA density, number, and the exact localization of the MRI foci might be considered as well. In addition, in a future prospective clinical trial a centralized pathology review and a centralized radiological review are necessary.

One of the most recent molecular diagnostic tests described is the STHLM3 model [35]. This model combines several plasma protein biomarkers, genetic polymorphisms, and clinical information. The STHLM3 model could identify a population where the tumor detection rate reached more than 50% [35]. Nevertheless, in our highly selected patient cohort, our mathematical model correctly predicted a PCa diagnosis in 72% of cases.

## 5. Conclusions

In summary, our findings demonstrate that the addition of miRNA expression information to MRI imaging and clinical data is able to enhance the tumor prediction rate. Particularly for patients with PI-RADS ≤3 lesions, the miRNA-supported predictive models perform very well at predicting a clinically significant PCa.

## Figures and Tables

**Table 1:** Patients’ characteristics and univariate association of individual variables with clinical end points.

Parameter		Discovery Cohort (*n* = 80)	Validation Cohort (*n* = 209)	Complete Cohort (*n* = 289)	Clinical end Point: PCa P; Relative Risk (95%CI)	Clinical end Point: Significant PCa P; Relative Risk (95%CI)
Patient age; median (IQR)		66 (59.5–72.25)	65 (60–71)	66 (60–72)	<0.01 1.08 (1.05–1.21)	<0.01 1.08 (1.05–1.12)
Pre-biopsy PSA level (ng/mL; median, IQR)		8.2 (6.8–12.5)	8.2 (6.0–13.2)	8.19 (6.1–13.1)	<0.01 1.06 (1.03–1.07)	<0.01 1.08 (1.04–1.13)
Previous biopsy; N (%)					0.35 1.28 (0.76–2.17)	0.22 1.39 (0.82–2.35)
	No	34 (42.5)	44 (21.0)	78 (27.0)		
	Yes	46 (57.5)	165 (79.0)	211 (73.0)		
Highest PI-RADS score; N (%)					<0.01 2.07 (1.55–2.79)	<0.01 2.39 (1.76–3.33)
	1	1 (1.3)	0 (0)	1 (0.4)		
	2	0 (0)	24 (11.5)	24 (8.3)		
	3	8 (10.0)	57 (27.3)	65 (22.5)		
	4	49 (61.2)	84 (40.2)	133 (46.0)		
	5	22 (27.5)	44 (21.0)	66 (22.8)		
Biopsy Gleason score (GS); N (%)					n.c.	n.c.
	Tumor free	38 (47.5)	103 (49.3)	141 (48.8)		
	6	9 (11.2)	17 (8.1)	26 (9.0)		
	7a (GS3+4)	16 (20.0)	49 (23.4)	65 (22.5)		
	7b (GS4+3)	9 (11.2)	9 (4.3)	18 (6.2)		
	8	2 (2.5)	11 (5.3)	13 (4.5)		
	9	5 (6.3)	20 (9.6)	25 (8.7)		
	10	1 (1.3)	0 (0)	1 (0.3)		
Targeted biopsy cores; median (IQR)		3 (2–4)	6 (4–7)	5 (3–6)	0.12 0.93 (0.85–1.03)	0.98 0.98 (0.89–1.08)
Systematic biopsy cores; median (IQR)		9 (8–11)	12 (12–12)	12 (11–12)	0.49 0.99 (0.91–1.08)	0.80 0.99 (0.91–1.08)
Tumor status; N (%)					n.c.	n.c.
	No tumor	36 (45.0)	103 (49.3)	139 (48.1)		
	Tumor	44 ^1^ (55.0)	106 (50.7)	150 ^1^ (51.9)		
ΔCt miR-141-3p; median (IQR)		18.4 (15.2–18.4)	16.7 (15.0–19.6)	16.9 (15.1–20.3)	0.46 1.03 (0.96–1.10)	0.97 1.00 (0.93–1.08)
ΔCt miR-375-5p; median (IQR)		16.5 (14.4–19.1)	14.7 (13.4–19.2)	15.3 (13.6–19.2)	0.31 1.04 (0.97–1.11)	0.92 0.99 (0.93–1.06)
ΔCt miR-21-5p; median (IQR)		9.2 (8.8–9.8)	7.4 (7.0–8.0)	7.9 (7.2–8.8)	0.74 1.03 (0.87–1.21)	0.61 0.96 (0.80–1.13)
ΔCt miR-320b; median (IQR)		9.8 (8.6–10.7)	10.2 (9.8–10.7)	10.2 (9.8–10.7)	0.18 0.89 (0.74–1.05)	0.18 0.88 (0.74–1.05)
ΔCt miR-210-3p; median (IQR)		12.8 (12.3–14.8)	12.7 (11.9–13.7)	12.7 (12.1–13.8)	0.36 0.97 (0.89–1.04)	0.22 0.95 (0.88–1.03)
ΔCt let-7c-5p; median (IQR)		11.1 (10.7–11.5)	11.7 (11.1–12.5)	11.5 (11.0–12.2)	0.14 1.12 (0.97–1.32)	0.39 1.07 (0.92–1.23)
ΔCt miR-486-5p; median (IQR)		3.2 (2.8–3.5)	3.1 (2.8–3.4)	3.1 (2.8–3.5)	0.482 1.15 (0.78–1.79)	0.70 0.92 (0.60–1.37)

IQR—interquartile range; ^1^ Two patients with a tumour-free biopsy had histologically confirmed prostate cancer; n.c.—not calculated.

**Table 2:** Predictive performance of clinical parameters and miRNAs in the discovery and validation cohorts.

Model Establishment: Discovery Cohort						
Parameters of the Model						
Clinical parameters	miRNA	PPV (%)	NPV (%)	Sensitivity (%)	Specificity (%)	AUC
Age, PSA, Prev. biopsy, PI-RADS		66.0	66.7	79.5	50.0	0.684
Age, PSA, Prev. biopsy, PI-RADS	miR-141-3p	64.8	65.3	79.5	52.7	0.684
Age, PSA, Prev. biopsy, PI-RADS	miR-375-3p	64.8	65.3	79.5	52.8	0.689
Age, PSA, Prev. biopsy, PI-RADS	miR-21-5p	68.0	66.7	77.3	55.5	0.718
Age, PSA, Prev. biopsy, PI-RADS	miR-320b	64.1	62.9	77.3	52.8	0.681
Age, PSA, Prev. biopsy, PI-RADS	miR-210-3p	67.3	67.8	79.5	52.8	0.689
Age, PSA, Prev. biopsy, PI-RADS	let-7c-5p	86.0	68.7	77.3	61.1	0.707
Age, PSA, Prev. biopsy, PI-RADS	miR-486-5p	70.8	66.7	77.3	55.5	0.697
**Model Validation: Validation Cohort**						
**Parameters of the Model**						
**Clinical parameters**	**miRNA**	**PPV (%)**	**NPV (%)**	**Sensitivity (%)**	**Specificity (%)**	**AUC**
Age, PSA, Prev. biopsy, PI-RADS		65.4	65.6	67.9	63.1	0.732
Age, PSA, Prev. biopsy, PI-RADS	miR-141-3p	66.7	60.3	67.9	65.0	0.734
Age, PSA, Prev. biopsy, PI-RADS	miR-375-3p	68.1	68.7	70.8	66.0	0.736
Age, PSA, Prev. biopsy, PI-RADS	miR-21-5p	75.6	54.6	26.4	91.3	0.702
Age, PSA, Prev. biopsy, PI-RADS	miR-320b	69.3	66.7	66.0	66.7	0.737
Age, PSA, Prev. biopsy, PI-RADS	miR-210-3p	65.1	65.9	68.9	62.1	0.733
Age, PSA, Prev. biopsy, PI-RADS	let-7c-5p	63.8	66.7	71.7	58.3	0.734
Age, PSA, Prev. biopsy, PI-RADS	miR-486-5p	65.7	58.7	65.1	65.0	0.719

**Table 3:** Comparison of pathological and predicted outcome; covariates and regression coefficients of the regression model for predicting significant PCa in patients with PI-RADS scores 1, 2, and 3. For binomial classification, the optimized threshold of 14% risk was used.

		Predicted Disease Status		
		Tumor-free/Gleason 6	Gleason 7a–10	
Pathological disease status	Tumor-free/Gleason 6 *	33	35	Specificity 48.5%
	Gleason 7a–10 *	1	20	Sensitivity 95.2%
		NPV 97.1%	PPV 36.4%	
Covariate	Model coefficient			
Intercept	−6.47144586			
Patient age	0.06001647			
Pre-biopsy PSA level	0.09607283			
Previous biopsy procedure	−1.36328590			
miR-486-5p	−0.52307176			
let-7c-5p	0.30509631			

* Tumor-free/Gleason 6 corresponding to Tumor-free/Grade group 1; Gleason 7a-10 corresponding to Grade groups ≥2.

## Data Availability

Data is contained within this article and the Supplementary Materials. Further data can be obtained from the corresponding author upon reasonable request.

## Supplementary Materials

The following are available online at https://www.mdpi.com/article/10.3390/cells10061315/s1, Figure S1: Calibration plot of the optimized regression model for predicting tumor status; Figure S2: Calibration plot of the optimized regression model for predicting significant PCa in patients with PI-RADS scores 1, 2, and 3; Figure S3: Receiver operator curve comparison. Table S1: Number of patients with corresponding Gleason scores and PI-RADS scores; Table S2: Comparison of pathological and predicted outcome; Covariates and regression coefficients of the optimized regression model for predicting tumor status; Table S3: Net expected regret differences at defined threshold cut-off values. Pair-wise comparisons of regret differences for the detection of significant PCa in patients with lesions of PI-RADS ≤3.
