# Supplementary material for: Serum miRNAs Support the Indication for MRI-Ultrasound Fusion-Guided Biopsy of the Prostate in Patients with Low-PI-RADS Lesions
Source: Cells. 2021 May 25;10(6):1315. doi: 10.3390/cells10061315 (PMC8226644; doi:10.3390/cells10061315)
Supplement: Supplementary file 1 [file cells-10-01315-s001.zip › Supplementary material.pdf]

Serum miRNAs support the indication for transrectal MRI-ultrasound fusion-guided biopsy of the prostate in patients with low-PI-RADS lesions

Bastian Keck, Angelika Borkowetz, Julia Poellmann, Thilo Jansen, Susanne Fuessel, Andreas Kahlmeyer, Manfred Wirth, Johannes Huber, Alexander Cavallaro, Matthias Hammon, Ivan Platzek, Arndt Hartmann, Gustavo Baretton, Frank Kunath, Danijel Sikic, Helge Taubert, Bernd Wullich, Kati Erdmann, Sven Wach

Table S1. Number of patients with corresponding Gleason scores and PI-RADS scores.

(a): Discovery cohort					
**Parameter**	**PI-RADS 1**	**PI-RADS 2**	**PI-RADS 3**	**PI-RADS 4**	**PI-RADS 5**
Detected Tumor Gleason Score					
Negative Biopsy (N=38)	0	0	6	25	7
6 (N=9)	0	0	1	6	2
7a (N=16)	1	0	0	10	5
7b (N=9)	0	0	0	7	2
8 (N=2)	0	0	1	0	1
9 (N=5)	0	0	0	1	4
10 (N=1)	0	0	0	0	1
(b): Validation cohort					
Parameter	PI-RADS 1	PI-RADS 2	PI-RADS 3	PI-RADS 4	PI-RADS 5
Detected Tumor Gleason Score					
Negative Biopsy (N=103)	0	16	38	43	6
6 (N=17)	0	3	5	7	2
7a (N=49)	0	1	9	25	14
7b (N=9)	0	1	1	2	5
8 (N=11)	0	3	1	5	2
9 (N=20)	0	0	3	2	15
10 (N=0)	0	0	0	0	0

Table S2. Comparison of pathological and predicted outcome; Covariates and regression coefficients of the optimized regression model for predicting Tumour status.

		**Predicted disease status**		
		Tumor-free	Tumor	
**Pathological disease status**	Tumor-free	94	44	Specificity 68.1%
	Tumor	43	107	Sensitivity 71.3%
		NPV 68.6%	PPV 70.9%	
**Covariate**	**Model coefficient**			
Intercept	10.01852936			
Patient age	-0.07036056			
Pre-biopsy PSA level	-0.03517680			
Previous biopsy procedure	-0.22310466			
Highest PIRADS score	-0.63490785			
miR-375-3p	-0.03835900			
let-7c-5p	-0.14499602			
miR-210-3p	0.02283841			
miR-486-5p	-0.12069582			

Table S3. Net. expected regret differences at defined threshold cut-off values. Pair-wise comparisons of regret differences for the detection of significant PCa in patients with lesions of PI-RADS ≤3.

Threshold	Biopsy none	Advantage Biopsy all over none	Advantage model over biopsy none	Advantage model over biopsy all
0.01	0	0.228237430	0.22823743	NA
0.02	0	0.220362302	0.22105022	0.0006879156
0.03	0	0.212324800	0.21371482	0.0013900151
0.04	0	0.204119850	0.20739700	0.0032771536
0.05	0	0.195742164	0.19041987	-0.0053222945
0.06	0	0.187186230	0.18312216	-0.0040640689
0.07	0	0.178446297	0.17905038	0.0006040836
0.08	0	0.169516365	0.17293600	0.0034196385
0.09	0	0.160390172	0.16804544	0.0076552661
0.10	0	0.151061174	0.16604245	0.0149812734
0.11	0	0.141522535	0.16083828	0.0193157430
0.12	0	0.131767109	0.15883555	0.0270684372
0.13	0	0.121787421	0.15924060	0.0374531835
0.14	0	0.111575647	0.16070029	0.0491246407
0.15	0	0.101123596	0.14606742	0.0449438202
0.16	0	0.090422686	0.13376137	0.0433386838
0.17	0	0.079463923	0.12197103	0.0425071071
0.18	0	0.068237873	0.12441765	0.0561797753
0.19	0	0.056734637	0.10861423	0.0518795950
0.20	0	0.044943820	0.10393258	0.0589887640
0.21	0	0.032854501	0.10809273	0.0752382307
0.22	0	0.020455200	0.10688562	0.0864304235
0.23	0	0.007733839	0.09805924	0.0903254049
0.24	0	-0.005322295	0.08633944	0.0916617386
0.25	0	-0.018726592	0.08988764	0.1086142322
0.26	0	-0.032493167	0.08624355	0.1187367142
0.27	0	-0.046636909	0.07126366	0.1179005695
0.28	0	-0.061173533	0.05617978	0.1173533084
0.29	0	-0.076119639	0.05681279	0.1329324260
0.30	0	-0.091492777	0.05778491	0.1492776886
0.31	0	-0.107311513	0.05406286	0.1613743690
0.32	0	-0.123595506	0.06080635	0.1844018506
0.33	0	-0.140365588	0.05718598	0.1975515680
0.34	0	-0.157643854	0.05447736	0.2121212121
0.35	0	-0.175453760	0.04062230	0.2160760588
0.36	0	-0.193820225	0.05688202	0.2507022472
0.37	0	-0.212769752	0.04369538	0.2564651329
0.38	0	-0.232330555	0.04168177	0.2740123233
0.39	0	-0.252532695	0.03960214	0.2921348315
0.40	0	-0.273408240	0.04494382	0.3183520599
0.41	0	-0.294991430	0.04741954	0.3424109693
0.42	0	-0.317318869	0.04610616	0.3634250291

0.43	0	-0.340429726	0.05322295	0.3936526710
0.44	0	-0.364365971	0.06099518	0.4253611557
0.45	0	-0.389172625	0.06026558	0.4494382022
0.46	0	-0.414898044	0.04827299	0.4631710362
0.47	0	-0.441594234	0.03625185	0.4778460886
0.48	0	-0.469317200	0.03543647	0.5047536733
0.49	0	-0.498127341	0.03458912	0.5327164574
0.50	0	-0.528089888	0.03370787	0.5617977528
0.51	0	-0.559275396	0.03279064	0.5920660399
0.52	0	-0.591760300	0.03183521	0.6235955056
0.53	0	-0.625627540	0.03083911	0.6564666507
0.54	0	-0.660967269	0.01856375	0.6795310210
0.55	0	-0.697877653	0.01747815	0.7153558052
0.56	0	-0.736465781	0.01634321	0.7528089888
0.57	0	-0.776848707	0.01515547	0.7920041808
0.58	0	-0.819154628	0.01391118	0.8330658106
0.59	0	-0.863524253	0.02877501	0.8922992601
0.60	0	-0.910112360	0.02808989	0.9382022472
0.61	0	-0.959089600	0.01613368	0.9752232786
0.62	0	-1.010644589	0.01537552	1.0260201064
0.63	0	-1.064986335	0.01457637	1.0795627088
0.64	0	-1.122347066	0.01373283	1.1360799001
0.65	0	-1.182985554	0.01284109	1.1958266453
0.66	0	-1.247191011	0.02247191	1.2696629213
0.67	0	-1.315287709	0.02247191	1.3377596187
0.68	0	-1.387640449	0.02247191	1.4101123596
0.69	0	-1.464661109	0.02247191	1.4871330192
0.70	0	-1.546816479	0.02247191	1.5692883895
0.71	0	-1.634637737	0.02247191	1.6571096474
0.72	0	-1.728731942	0.02247191	1.7512038523
0.73	0	-1.829796088	0.02247191	1.8522679983
0.74	0	-1.938634399	0.02247191	1.9611063094
0.75	0	-2.056179775	0.02247191	2.0786516854
0.76	0	-2.183520599	0.02247191	2.2059925094
0.77	0	-2.321934538	0.02247191	2.3444064485
0.78	0	-2.472931563	0.02247191	2.4954034729
0.79	0	-2.638309256	0.02247191	2.6607811664
0.80	0	-2.820224719	0.02247191	2.8426966292
0.81	0	-3.021289178	0.02247191	3.0437610881
0.82	0	-3.244694132	0.02247191	3.2671660424
0.83	0	-3.494382022	0.02247191	3.5168539326
0.84	0	-3.775280899	0.02247191	3.7977528090
0.85	0	-4.093632959	0.02247191	4.1161048689
0.86	0	-4.457463884	0.02247191	4.4799357945
0.87	0	-4.877268799	0.02247191	4.8997407087
0.88	0	-5.367041199	0.02247191	5.3895131086

0.89	0	-5.945863126	0.02247191	5.9683350358
0.90	0	-6.640449438	0.02247191	6.6629213483
0.91	0	-7.489388265	0.02247191	7.5118601748
0.92	0	-8.550561798	0.02247191	8.5730337079
0.93	0	-9.914927769	0.02247191	9.9373996790
0.94	0	-11.734082397	0.02247191	11.7565543071
0.95	0	-14.280898876	0.02247191	14.3033707865
0.96	0	-18.101123596	0.02247191	18.1235955056
0.97	0	-24.468164794	0.01123596	24.4794007491
0.98	0	-37.202247191	0.00000000	0.0000000000
0.99	0	-75.404494382	0.00000000	0.0000000000

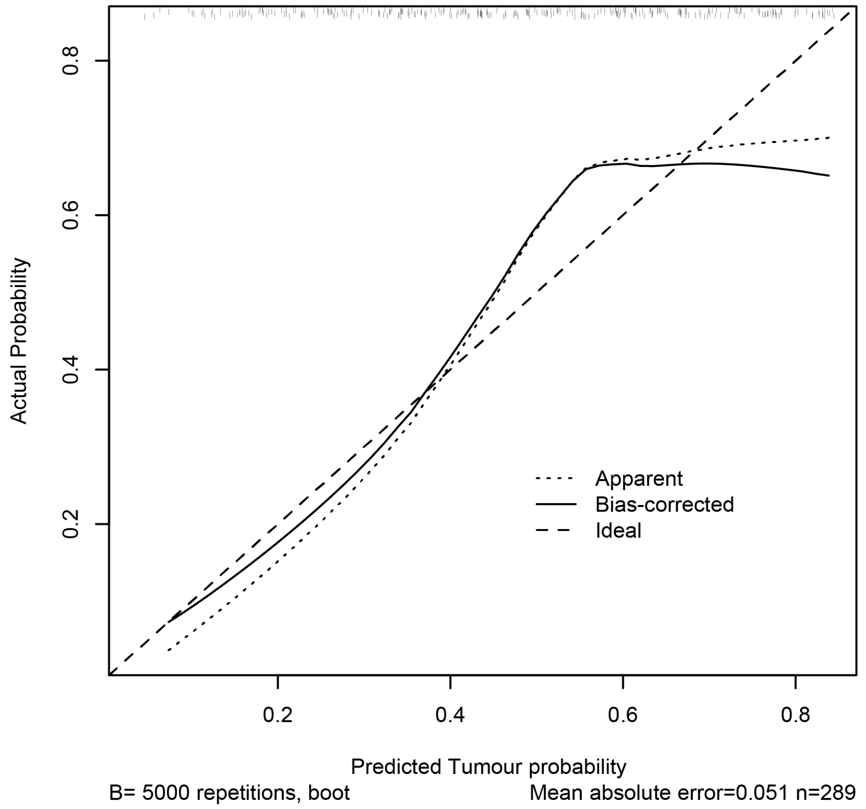

Supplementary Fig 1. Calibration plot of the optimized regression model for predicting Tumour status. Predicted and actual tumor probability, based on 5000 bootstrap replicates.

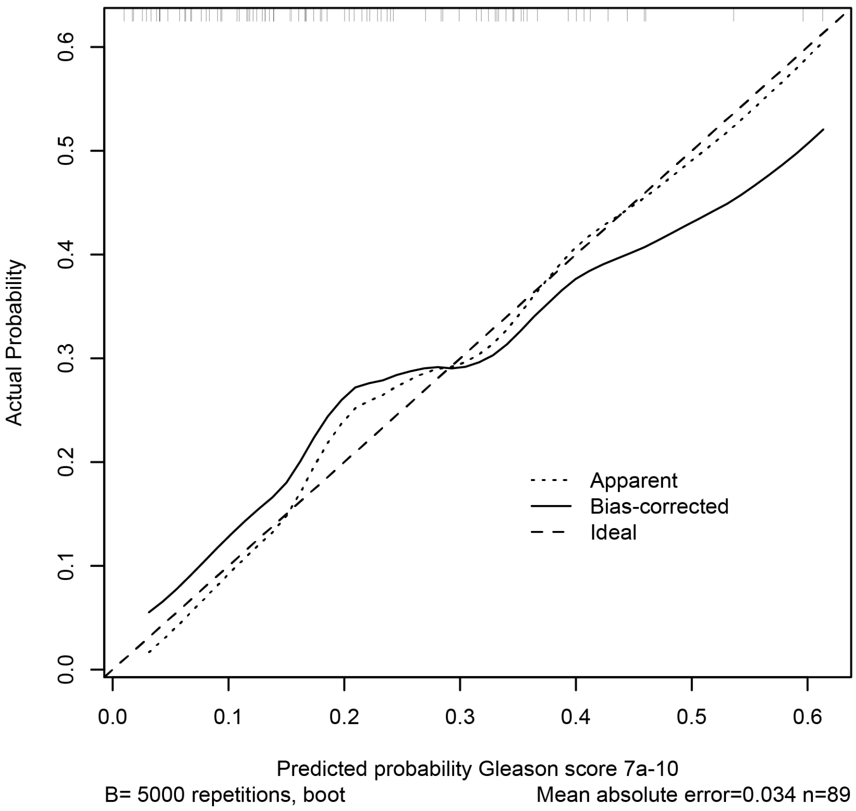

Supplementary Fig 2. Calibration plot of the optimized regression model for predicting significant PCa in patients with PI-RADS scores 1, 2, and 3. Predicted and actual probability of significant PCa, based on 5000 bootstrap replicates.

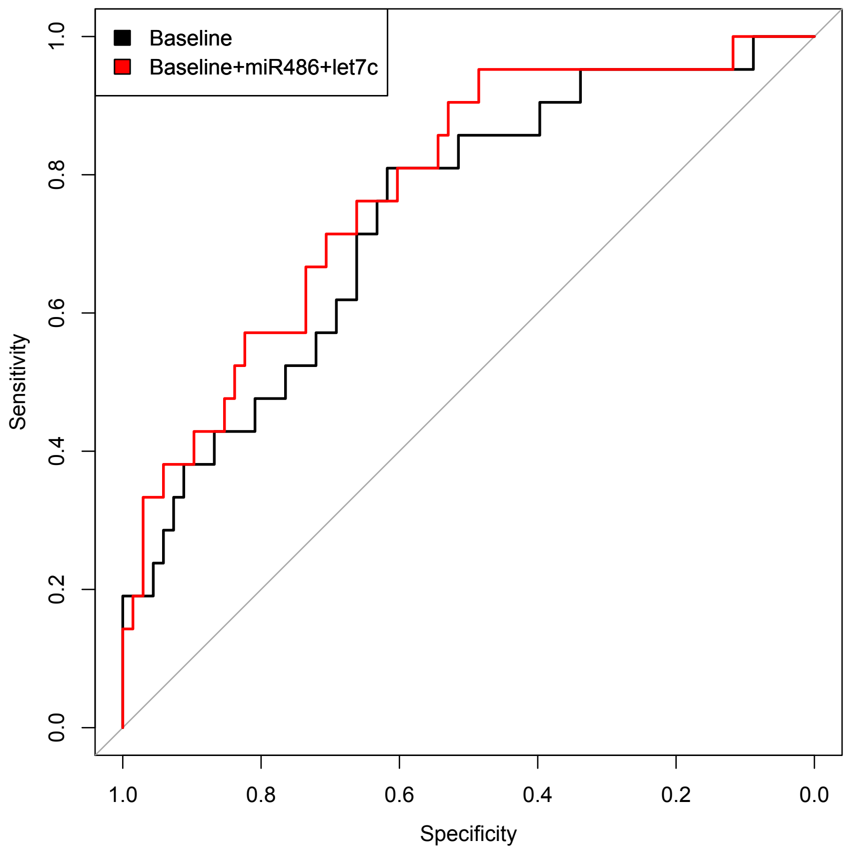

Supplementary Fig3. Receiver-operator curve comparison of the baseline model and the baseline model with miRNAs miR-486-5p and let-7c-5p for the prediction of csPCa.
